# Per operative discovery of Placenta Praevia Percreta: a case report

**DOI:** 10.11604/pamj.2013.16.53.1912

**Published:** 2013-10-15

**Authors:** Lawrence Mbuagbaw, Frederick Lifangi-Ikomi Morfaw

**Affiliations:** 1 Centre for the Development of Best Practices in Health (CDBPH), Yaoundé Central Hospital, Avenue Henri Dunant, Messa, PO Box 87, Yaoundé, Cameroon; 2 Department of Obstetrics and Gynaecology, Faculty of Medicines and Biomedical Sciences, University of Yaoundé 1, PO Box 1364, Yaoundé, Cameroon

**Keywords:** Placenta Praevia Percreta, caesarian section, vaginal bleeding

## Abstract

Placenta percreta is a rare pathological entity with challenging diagnostic and therapeutic requirements especially for resource limited settings. We present here the case of a 40 year old woman with a per operative diagnosis of placenta accreta during a caesarian section indicated for placenta praevia. We highlight the diagnostic and therapeutic difficulties associated with this condition especially in low resource settings. Physicians performing caesarian sections should be prepared for complex intra-operative findings in high risk patients.

## Introduction

Placenta accreta is a general term that describes any placenta that is abnormally firmly adherent to the uterine wall [1]. Three forms exist depending on the depth of penetration, namely; placenta accreta, placenta increta and placenta percreta. Placenta accreta refers to a placenta that is firmly adherent to the uterine wall without myometrial penetration. Placenta increta refers to a placenta that partially invades the myometrium, and placenta percreta refers to a full thickness penetration of the myometrium and serosa by the placenta, with occasional invasion of adjacent organs such as the bladder [1]. It is a rare condition, with a reported incidence of about 1 in 2500 pregnancies [2]. It is potentially life threatening because it can lead to catastrophic blood loss and death [3]. Antenatal diagnosis is required to plan delivery and minimize complications [3]. Ultrasound or Magnetic Resonance Imaging (MRI) are useful in establishing antenatal diagnosis [1], yet in resource limited settings, these tools are not readily available. We present here a case of placenta percreta discovered during a caesarian section in a 40 year old patient, indicated for haemorrhagic placenta praevia. We also discuss the diagnostic and therapeutic difficulties for this condition in resource limited settings.

## Patient and observation

A 40 year old woman gravida 8 Para 7, at 39 weeks and 2 days gestation presented at our antenatal care clinic with painless per vaginal bleeding of sudden onset. She had previously had 7 normal term deliveries of which the biggest baby weighed 3700 grams. During her seventh delivery 8 years prior, she suffered from placental retention, but we found no records of the procedures performed. Only one antenatal care visit had been attended during this pregnancy.

On admission, the patient was haemodynamically stable. The fundal height was 34cm, and there were no uterine contractions. The presenting part was difficult to appreciate. The foetal heart rate was 145 beats per minute. Initial vaginal examination was limited to vulvar inspection which revealed fresh blood on the vulva. An ultrasound examination revealed a single intra-uterine live female fetus in a breech presentation and a type II placenta praevia. Given this finding, and in the presence of the active bleeding, an emergency caesarian section was performed under general anesthesia.

**Figure 1:**
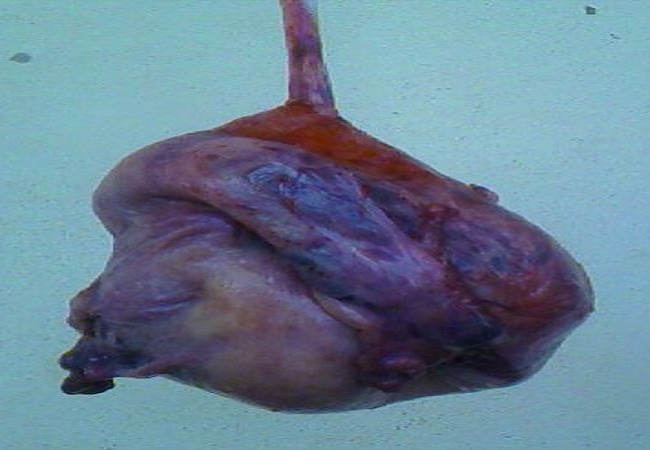
Uterus dangling from still attached placenta and umbilical cord

The per operative findings were a gravid uterus with whitish fibrous strands on the external surface of the lower uterine segment invading the serosa, but without extension to the bladder. Hysterotomy was performed by corporeal incision. We extracted a live female baby weighing 3600g, with an APGAR score of 7 and 8 at the 1^st^ and 5^th^ minutes respectively. Part of the placenta was in the lower uterine segment and almost completely embedded in the myometrium and serosa, with less than 40% in the uterine cavity. Manual placental extraction was impossible. We performed a subtotal hysterectomy to remove the entire zone of placental infiltration (Figure 1). We obtained satisfactory haemostasis. The estimated blood loss including amniotic fluid was about 2000cc, and the patient received 2 pints of blood during surgery.

The post-operative evolution was uneventful. The patient was discharged seven days later with a blood pressure of 100/70mmHg, a pulse of 80 beats per minute and a temperature of 37.0 Celsius. The surgical wound was clean, dry and well apposed.

## Discussion

Placenta accreta is thought to be due to the partial or total absence of the decidua basalis[4]. Failure of the decidua basalis layer to reconsititute often occurs after a caesarian section[5]. Other risk factors include advanced maternal age, abnormal placentation and a history of dilatation and curettage [6]. Our patient was 40 years old and had an associated abnormal placentation (placenta praevia). Her history of placental retention was also suggestive of endo-uterine manipulation to evacuate the uterus. Hence our patient was definitely at risk for this condition.

Antenatal diagnosis requires imaging using either ultrasound or MRI since the condition is usually asymptomatic before delivery [1]. In a center without ultrasound facilities a pre delivery diagnosis would be impossible. This is usually the case in resource limited settings, and the diagnosis is often made intrapartum or per operatively. In our case, an ultrasound was done to investigate third trimester bleeding. The presence of a placenta praevia was sufficient for us to envisage surgical management. However, prior knowledge of the underlying condition would have permitted us to take additional precautions in terms of blood products, duration of surgery and surgical skill evaluation.

The generally accepted therapeutic approach is a planned preterm caesarian hysterectomy, leaving the placenta in situ given that attempted delivery usually results in massive hemorrhage [1]. Emergency caesarian section without adequate preparation is not advisable [1]. In our patient antepartum diagnosis was not established hence an emergency caesarian section was indispensable. During surgery, we performed a vertical incision on the uterus to deliver the infant while avoiding the placenta. We opted for a sub-total hysterectomy as this permitted rapid control of the hemorrhage and complete removal of the placenta.

It should however be noted that conservative management may be offered to a carefully selected number of patients desiring future fertility [1]. Attempts at conservative management should be mindful of the fact that the results are highly unpredictable, and the chances of subsequent successful pregnancies mediocre [7]. One conservative approach involves ligation of the umbilical cord at the placental insertion and leaving the placenta in situ[8]. This is usually associated with postpartum administration of methotrexate to destroy the placenta. A recent committee opinion did not however recommend the use of methotrexate in the postpartum management of placenta accrete [9]. Both hysterectomy and conservative management require a reasonable amount of skill and experience, which may not be present in low- resource settings.

## Conclusion

This case highlights the need for sonography in women at risk for placental infiltration (advanced age, abnormal placentation and uterine manipulation) and accrued vigilance and preparedness when placenta praevia is detected. For poorly equipped centers with limited diagnostic facilities, the discovery of an abnormally deep insertion of the placenta should be treated as a surgical emergency. Considerable surgical skill is required to rapidly switch from a simple caesarian section to a caesarian hysterectomy. General practitioners working in ill-equipped settings should be able to handle more surgical emergencies than the caesarian section and consider placenta percreta when performing surgery on women at risk.
